# The effect of visual attention process and thinking styles on environmental aesthetic preference: An eye-tracking study

**DOI:** 10.3389/fpsyg.2022.1027742

**Published:** 2023-01-16

**Authors:** Wan Chen, Rongbin Ruan, Weiwei Deng, Junxi Gao

**Affiliations:** ^1^School of Economics and Management, Fuzhou University, Fuzhou, China; ^2^School of Tourism and Culture Industry, Sichuan Tourism University, Chengdu, China

**Keywords:** thinking styles, visual attention process, top–down process, bottom–up process, environmental aesthetic preference

## Abstract

People often form different aesthetic preferences for natural and built environments, which affects their behavioral intention; however, it remains unknown whether this difference in aesthetic preference is due to differences in thinking styles. However, whether tourists’ aesthetic preferences differ when using different visual attention processes has not been studied further. This study used eye-tracking and self-reporting to investigate these questions. The results show that natural environment images are more favored visually because they can evoke in tourists larger pupil diameters and longer scan paths, but we found no significant difference in fixation duration and fixation counts. We also found that the scanning path of tourists who predominantly rely on intuitive thinking is modulated by the bottom-up attention process, while the scanning path of tourists who prefer rational thinking is modulated by the top-down attention process. In the bottom-up process, tourists who prefer rational thinking exhibit more positive aesthetic preferences and emotional arousal. In summary, the present study verified that aesthetic preference is more likely to be influenced by both thinking style and visual attention processing. The results of the present work provide preliminary evidence that the aesthetic preference of the environment is not only related to visual attention but also affected by the individual visual attention process and thinking style.

## Introduction

The environmental aesthetics literature suggests a higher likability for natural over built scenes, which also holds true for tourism images (Twedt et al., 2019). In tourism research, many scholars have divided travel destinations into natural and built environments (Sparks and Wang, 2014). Previous studies have shown that compared with the built environment, the natural environment attracts more visual attention. This is manifested by the eye movement index: longer fixation duration and greater fixation counts. Therefore, the natural environment image attracts more visual attention, suggesting that the image has a high viewer preference (Deng et al., 2021). Nature is seen as highly compatible with human purposes and tendencies, and the soft “charm” elements of nature (e.g., walks in forests and patterns of clouds) can attract attention effortlessly and provide the brain with opportunities to engage in reflection (Sparks and Wang, 2014). Notably, these studies did not find an association between looking and attention (Wang and Sparks, 2016). However, it is commonly believed that people look longer at stimuli they like (Motoki et al., 2021). Therefore, studies on the association between preference and attention are still insufficient, which affects people’s understanding of landscape preferences and distribution of attention.

According to psychology and marketing, it is widely considered that there are two different ways of thinking that influence consumer decisions: one of them is intuitive, natural, heuristic, automatic, schematic, prototypical, narrative, implicit, imagistic-nonverbal, experiential, mythical, and called the first-signal system. The other is thinking-conceptual-logical, analytical-rational, deliberative-effortful-intentional-systematic, explicit, extensional, verbal, and logos, and is named the second-signal system (Epstein et al., 1996; Wang et al., 2017). In our daily lives, most of our choices are made through intuitive thinking without careful consideration (Kahneman, 2003). Cognitive-experiential self-theory (CEST) proposes that a rational system functions mostly at the conscious level and is intentional, analytical, mainly verbal, and relatively affect-free. Intuitive systems are thought to be automatic, preconscious, holistic, associative, chiefly nonverbal, and intimately associated with affect (Witteman et al., 2009; Phillips et al., 2016). Epstein et al. (1996) believed that people differ in how they go about decision-making, mainly owing to the way in which they are more inclined toward. For example, mathematical problems tend to require rational thinking, whereas interpersonal problems tend to use intuitive thinking. In addition, information based on emotions and personal experience is more effective for people who mainly depend on intuitive thinking. In contrast, information based on facts and logical arguments could be more appealing to those who attach more importance to rational thinking (Ares et al., 2014; Alaybek et al., 2022).

In terms of research on tourists’ visual attention, some scholars have confirmed the differences in visual attention among tourists of different nationalities (Wang and Sparks, 2016). Different thinking styles differ considerably in terms of visual gaze, information search, and target selection. They found that rational consumers attract longer fixation durations than intuitive consumers (Ares et al., 2014; Motoki et al., 2021). However, the individual rational and intuitive thinking styles in assessing aesthetic preferences are not well understood. It is important to note that the difference in the extent to which people rely on the two thinking styles can have implications for understanding the aesthetic preferences of different types of tourism images.

Top-down and bottom-up control of attention, which refers to endogenous and exogenous attention, respectively, are usually defined as goal-driven attention and stimulus-driven attention (Orquin et al., 2013). Bottom-up attention mechanisms are reflexive, automated, and unconscious. Consumers automatically pay attention to regions with higher visual salience in the object’s visual context. Factors like size, color, and layout affect attention distribution through the bottom-up control of attention. The top-down attention mechanism is subconsciously driven with higher cognition. Factors like information processing purposes, involvement, motivations, familiarity, and so on affect consumers’ attention distribution through top-down control of attention (Van der Lans and Wedel, 2017). Visual marketing attention theory (Wedel and Pieters, 2008) considers top-down factors to be related to individuals’ unique characteristics, such as expectations, objectives, and emotions, which affect the process of attention. Bottom-up factors that affect attention come from the physical features of the visual stimuli to which consumers are exposed. Some studies found that consumers’ attention is driven by their personal interests (top–down) when there are no images, but an abundance of information is available on social platforms. However, when pictorial content is included, the visual attention paid by one’s eye gaze to online reviews increases (bottom-up; Bigne et al., 2020; Boardman and Mccormick, 2021).

The attentional process is associated with so-called downstream effects: preference formation, learning, choice, and eventually, sales (Wedel and Pieters, 2017). Moreover, there is an interaction between top-down and bottom-up visual attention, which can enhance attentional acquisition. On social media, there are many sources of information that our attention needs to be driven by personal interests (top-down). However, when there are pictures in the content, attention is clearly influenced by the pictures, which reflects bottom-up visual attention (Pieters and Wedel, 2004; Campos et al., 2020). In short, the assignment of visual attention is jointly determined by the properties of the target object and the goals and expectations of the observer (Folk et al., 1992). In short, the assignment of visual attention is jointly determined by the properties of the target object and goals and expectations of the observer. Previous studies have shown that more visual attention is likely to be paid to stimuli with a higher aesthetic preference (Tamás et al., 2021; Straffon et al., 2022; Xu and Shen, 2022). However, the relationship between top-down and bottom-up attentional processes and aesthetic preferences has not been discussed thoroughly.

The aim of this study was to evaluate the influence of visual attention processes and thinking styles on tourists’ aesthetic preferences and emotional arousal when evaluating destination images by employing eye tracking and self-reporting. This study seeks to answer the following questions: (1) Which type of destination image is more visually preferred when considering the influence of thinking styles on tourists’ aesthetic preferences? (2) What will happen in the aforementioned problem if the process of visual attention is considered? In particular, in the bottom-up process of aesthetic information processing, is there any difference in the influence of rational and intuitive thinking styles on tourists’ aesthetic preferences and the emotional arousal of destination images? Conversely, in the top-down process of aesthetic information processing, what are the differences between rational and intuitive thinking styles that affect tourists’ aesthetic preferences and emotional arousal of destination images? Theoretically, this study contributes to the literature by considering individual thinking style research on photographic tourism images and visual attention processing differences in response to aesthetic preference and emotional arousal to tourism images. Understanding how thinking styles and visual attention processing influence consumer aesthetic preference has interesting implications for the development and tailoring tourism images to specific markets. In the long run, our findings can provide guiding principles for destination managers to design more effective images to enhance that are more effective and more appealing to tourists’ visuals appeal and help to formulate a long-term program for targeted tourism marketing.

## Materials and methods

### Eye-tracking

Eye-tracking is a relatively new technique for studying visual attention and perception in tourism research. Based on the hypothesis that eye movement indicates the focus of a person’s attention. Many studies have shown that eye-tracking can more objectively evaluate visual effects than self-reports (Scott et al., 2019). The fixation duration and fixation count can indicate the focus of attention and provide information about what may be the most important in the scene. Furthermore, pupil size has a close relationship with emotions and can help predict subjective emotional experience (Alghowinem et al., 2014). Recent studies have shown that eye-tracking can capture objective and real-time data of visual appeal, which can be measured by fixation duration, fixation count, and pupil size (Cui et al., 2020). There was a noticeable change in pupil size when viewing both pleasant and unpleasant emotional images.

### Participants and groups

Sixty individuals participated in the study, of which 26 (43.3%) were women. Their ages ranged from 20 to 30 years old. Participants were recruited from among students in Fuzhou. All the participants had full-color vision and normal or corrected-to-normal vision. The participants signed an informed consent form before the experiment. The study complied with departmental ethics committee regulations. Before the experiment, the participants were asked to complete the Rational Experiential Inventory (REI) 31 (Epstein et al., 1996) to determine their dominant thinking style. The REI includes items within two dimensions: need for cognition (19 items) and faith in intuition (12 items). Participants were asked to score each item on a 5-point scale, ranging from 1 (completely disagree) to 5 (completely agree).

The SPSS 26.0 was used to process the REI. An exploratory factor analysis was used to verify the factor structure of the REI scale. Items with factor loadings higher than 0.5 were considered, and 21 effective items were ultimately valid. The internal reliability of each factor was tested using Cronbach’s alpha as shown in Appendix 1. The ideal number of clusters was selected as the solution that maximized the Bayesian information criterion (BIC). Analysis of variance (ANOVA) was performed on the two dimensions’ scores, considering the cluster as a fixed source of variation. The BIC showed that the best solution consisted of two clusters: Cluster 1 (mainly using rational thinking) consisted of 36 participants, and Cluster 2 (mainly using intuitive thinking) consisted of 24 participants (Table 1).

**Table 1:** Cluster analysis.

Model summary		Cluster distribution			Average profile
Algorithm	Cluster	Item	Number	Percentage (%)	
Two steps	Two clusters	Cluster1	36	60	0.8 (> 0.5, good)
		Cluster2	24	40	

### Preparation and procedure

Based on the types of landscape, images used in the eye-tracking study can be divided into two kinds: one of them is eight images of the natural environment and the other is eight images of the built environment. Images were downloaded from well-known travel websites and processed based on color and brightness. This study designed a two-factor experiment of 2 (landscape types: natural, built) × 2 (thinking modes: rational, intuitive) within a group. The instrument used in the experiment was a Hi-Speed Eye Tracking System manufactured by the SMI Company. Infrared light technology with a sampling frequency of 500 Hz was used to create reflection patterns on the corneas of participants’ eyes. The experimental design and the presence of stimulus materials were used in the experiment center software. The calibration of eyes and the date records of eye tracking were performed using iViewX3.5 software. Finally, the BeGaze 3.5 software was used to complete the extraction and analysis of eye-tracking data.

**Figure 1:**
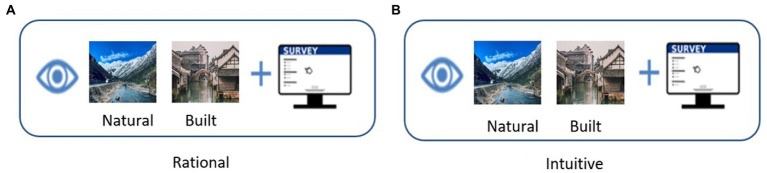
Procedure for eye-tracking research. J_usti. 2019 “Share a view from a previous trip with friends” Weibo, April 14, 2019 (https://weibo.com/5235197084/HpE4pnYlV). Reproduced with permission. Traveling_Lover. 2019. “Beautiful!” Weibo, April 12, 2019 (https://weibo.com/2628053771/HpkBrsp4m). Reproduced with permission.

The experimental process in this study consists of two parts. First, we investigated how participants perceived environmental aesthetics. In this session, we focused on the bottom-up process of visual attention in a freely browsing task. Second, we investigated how participants perceived environmental aesthetic in an aesthetic preference decision-making task. In this section, we focused on the top-down visual attention process. After the eye-tracking task, the computer presented 16 images once again. Each participant evaluated the images through a questionnaire on emotional arousal and their preference for images. Each image appeared with questions asking participants to rate their preference regarding images and arousal on a 7-point Likert scale. The measurement of preference, where 1 indicates least preferred/strongly disliked, and 7 indicates most preferred/strongly liked. The arousal measurement was obtained from Mano and Oliver (1993). SPSS 26.0 was used to analyze the variance. Sixteen images were presented randomly, and each stimulus was displayed until the participant responded. The eye-tracking research procedure is illustrated in Figure 1.

## Data analysis and results

For each dependent variable of fixation duration, fixation count, and pupil size, this study conducted a 2 (modes of thinking) × 2 (landscape types) mixed-design ANOVA. As shown in Table 2, the self-reported preference and arousal of images were analyzed in the same manner.

**Table 2:** Descriptive statistics.

Item	Modes of Thinking			
	Natural Environment		Built Environment	
	Rational	Intuitive	Rational	Intuitive
Fixation duration(s)	2.80 (0.16)	2.53 (0.27)	2.72 (0.28)	2.50 (0.12)
Fixation count	25.05 (2.68)	15.84 (3.17)	24.43 (1.95)	15.90 (1.63)
Pupil size (mm)	4.70 (0.24)	4.31 (0.24)	4.43 (0.28)	3.79 (0.27)
Preference	6.25 (0.58)	6.12 (0.55)	5.72 (0.49)	5.77 (0.56)
Arousal	5.98 (0.26)	5.94 (0.57)	4.72 (0.65)	4.70 (0.60)

### Eye-tracking data analysis

For each dependent variable (fixation duration, fixation count, and pupil size), this study conducted a 2 (modes of thinking) × 2 (landscape types) mixed-design variance (ANOVA) analysis. Levene’s test for the homogeneity of variance showed no significant differences. Table 3 summarizes the significant effects revealed by mixed ANOVA. The main effects of thinking modes on fixation duration, fixation count, and pupil size were significant (*p* < 0.001). The main effects of landscape types on pupil size was also significant (*p* < 0.001). However, the main effects of landscape types on fixation duration and count were not significant (*p* > 0.05). The interaction between landscape types and thinking modes was not significant (*p* > 0.05).

**Table 3:** Mixed-design analysis of variance.

Independent variable	Dependent variable		
	Fixation duration	Fixation count	Pupil size
Modes of thinking	*F* = 253.957	*F* = 241.272	*F* = 154.312
	*p* = 0.000^***^	*p* = 0.000^***^	*p* = 0.000^***^
Landscape type	*F* = 2.054	*F* = 1.092	*F* = 98.83
	*p* = 0.061	*p* = 0.078	*p* = 0.000^***^
Modes of thinking * type	*F* = 0.002	*F* = 0.243	*F* = 0.199
	*p* = 0.966	*p* = 0.624	*p* = 0.657

^***^p < 0.001.

### Self-report data analysis

As shown in Table 4, the main effects of landscape types on preference and arousal are significant (*p <* 0.001). However, the main effects of thinking modes on preference and arousal were not significant (*p* > 0.05). In addition, the interaction effect between landscape types and modes of thinking was not prominent (*p* > 0.05).

**Table 4:** Two-factor repeated measures analysis of variance for self-report.

Independent variable	Dependent variable	
	Preference	Arousal
Modes of thinking	*F* = 0.259	*F* = 0.038
	*p* = 0.613	*p* = 0.845
Landscape type	*F* = 17.756	*F* = 89.829
	*p* = 0.000^***^	*p* = 0.000^***^
Modes of thinking * type	*F* = 0.119	*F* = 0.342
	*p* = 0.732	*p* = 0.561

^***^p < 0.001.

It can be seen from the results of the self-report test (Table 2) that, compared with the built environment images, the natural environment images scored higher in preference and emotional arousal. Interaction with the natural environment can improve tourists’ emotional state, enhance concentration, relieve stress, and provide other restorative outcomes (Wang et al., 2019). Simultaneously, people prefer natural landscapes to building landscapes. This preference can be explained by the “biophilia effect,” which assumes that there exists a deep connection between humans and the nature. This is a biological need because a series of psychological benefits can be provided when people approach the benefits of nature. In addition, natural landscapes are more easily recalled than built-up landscapes (Sparks and Wang, 2014).

As noted earlier, tourists who mostly rely on rational thinking presented longer fixation durations, more fixation counts, and larger pupil sizes when facing natural environment images with a higher preference than tourists who mostly rely on intuitive thinking. However, we did not find that the natural environment images were significantly different from the built environment images in fixation duration and fixation counts. However, another study monitored the pupil diameter of testers when they viewed images to assess this effect. We found that tourists’ pupils were larger when viewing images of the natural environment. Kinner et al. (2017) found that people’s pupils became larger when they viewed pleasant emotional images. Therefore, we can speculate that pictures of natural environment images are more pleasurable for people, or that natural environment images are in general more favored.

### Scanpath analysis

**Figure 2:**
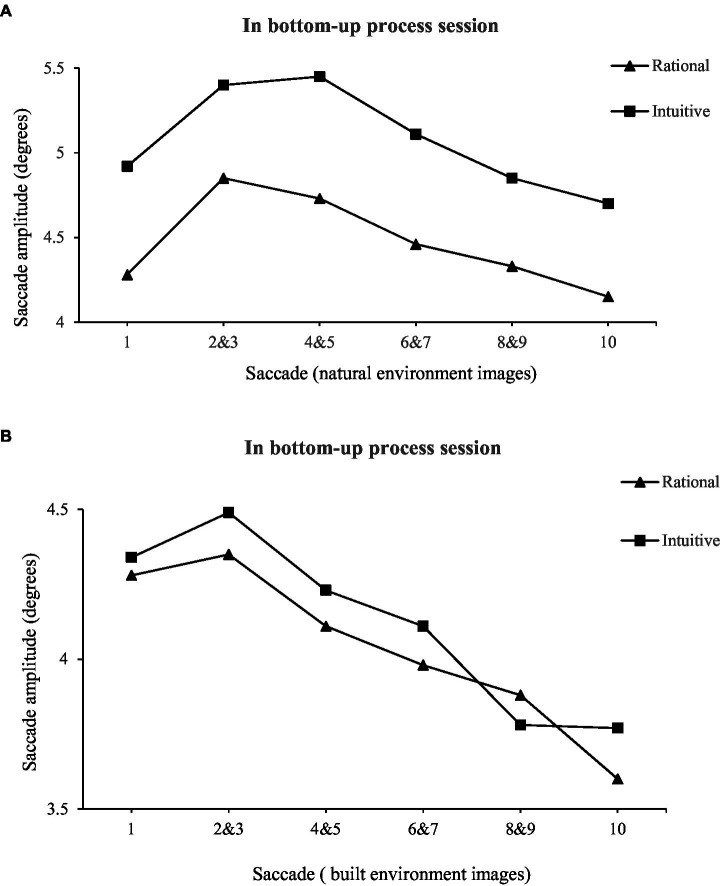
Saccade length initially increases and then decreases over the viewing interval in a freely browsing task.

**Figure 3:**
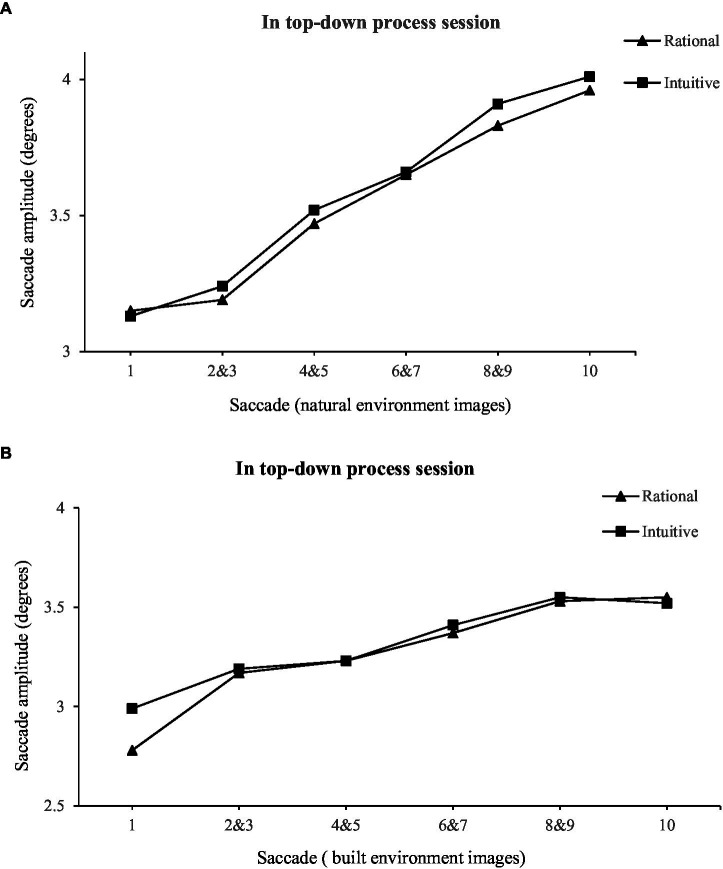
Saccade length initially increases over the viewing interval in aesthetic preference decision-making task.

As can be seen from Figures 2, 3 and Table 5, when participants performed a freely browsing task (bottom-up processes), compared with tourists with rational thinking (*M* = 64.7, SE = 2.1), tourists with intuitive thinking (*M* = 50.2, SE = 2.2) had longer saccade paths (*F* = 4.129, *p* < 0.05). When participants performed an aesthetic preference decision-making task (top-down processes), there was no significant (*F* = 1.049, *p* > 0.05) difference between the saccade path of tourists with intuitive thinking (*M* = 30.6, SE = 0.8) and those with rational thinking (*M* = 31.1, SE = 0.9). Contrarily, compared with participants performing an aesthetic preference decision-making task (top-down processes), the saccade path of tourists with intuitive thinking was longer when they performed a freely browsing task (bottom-up processes). The saccade path of tourists with rational thinking is shorter when performing aesthetic preference decision-making tasks than when performing freely browsing tasks.

**Table 5:** Total scan path (in degrees) for pictures viewed.

Environment	In bottom-up process session			In top-down process session		
	Natural	Built	Mean	Natural	Built	Mean
Rational	56.7 (2.1)	43.7 (2.3)	50.2 (2.2)	32.7 (0.9)	29.5 (0.8)	31.1 (0.9)
Intuitive	67.8 (2.0)	61.6 (2.9)	64.7 (2.1)	31.9 (0.8)	29.2 (1.0)	30.6 (0.8)

Standard errors are in parentheses.

People obtain information through their eyes and process it using their brains. Therefore, eye movements provide much information about the underlying cognitive processes. Scanpaths are an important tool for studying the sequential properties of eye movements and are characteristic of a given participant viewing a given pattern, which may reflect the temporal and spatial dynamics of underlying cognitive processing (Zhou et al., 2016).

Therefore, the saccade path of tourists with intuitive thinking becomes longer in bottom-up processes, and this study speculated that tourists with intuitive thinking are more susceptible to bottom-up factors. The saccade path of tourists with rational thinking becomes shorter in top-down processes, and this study speculated that tourists with rational thinking will be greatly influenced by top-down factors.

## Conclusions and discussion

### Discussion

The following conclusions were drawn by comparing eye-tracking and self-report data collected from rational and intuitive tourists when viewing travel photos. First, images of the natural environment are more visually preferred when considering the influence of thinking styles on tourists’ aesthetic preferences. In our experiments, natural environment images prompted a larger pupil diameter and a longer total scan path compared to built environment images. Specifically, rational and intuitive individuals both prompted a larger pupil diameter and longer scan paths when viewing natural environment images compared to built environment images. Although Wang and Sparks (2016) believed that higher arousal images and natural images attract a higher fixation count and a longer fixation duration than low-arousal and built images, there were no significant differences in the present study. Early aesthetic appreciation studies suggested that aesthetic preferences correlate with pupil size (Nagai and Georgiev, 2011). Kinner et al. (2017) found that people’s pupils became larger when they viewed pleasant images. Pupil size is closely related to emotions and can help predict subjective emotional experiences (Alghowinem et al., 2014). Generally speaking, the relationship between stimuli from the environment and behaviors caused by the environment can regulate emotions. Moreover, arousal may be the driving force of the decision-making process (Lin and Lo, 2016). In consumer behavior studies, a product with a higher preference causes a more positive emotional response (Tractinsky, 1997). Thus, this study speculated that emotional arousal may be closely related to individual cognitive processes and preferences. The natural environment images caused a higher preference for intuitive thinking individuals, thus leading to a more positive emotional response and larger pupil size.

In addition, previous research has shown that pleasurable content viewing elicits longer scan paths than when viewing complex content (Brunyé and Gardony, 2017). In the aesthetic field, preference is used to describe a pleasurable experience that requires individuals to estimate the visual properties of an object’s appearance. The visual system processes natural stimuli more easily and smoothly because the natural landscape may reduce cognitive load in favor of aesthetic estimation with lower cognitive demands. Compared with natural environment images, built environment images are less likely to attract observers’ visual attention (Wang et al., 2019) because the built environment could not provide much of a sense of “escape.”

Second, when participants performed a freely browsing task (bottom-up processes), compared to tourists with rational thinking, tourists with intuitive thinking demonstrated longer saccade paths. In contrast, when participants performed an aesthetic preference decision-making task (top-down processes), there was no significant difference between the saccade path of tourists with intuitive thinking tendencies and those with rational thinking tendencies. Conversely, compared with participants performing an aesthetic preference decision-making task (top-down processes), the saccade path of tourists with intuitive thinking was longer when participants performed a freely browsing task (bottom-up processes). The saccade path of tourists with rational thinking is shorter when performing aesthetic preference decision-making tasks than when performing freely browsing tasks. Combining above two conclusions, when the saccade path of tourists with intuitive thinking becomes longer in the bottom-up processes, it is inferred that tourists with intuitive thinking tendencies are more susceptible to bottom-up factors. The saccade path of tourists with rational thinking becomes shorter in the top-down attention process, and it is inferred that tourists with rational thinking are greatly modulated by the top-down attention process. In terms of aesthetic evaluation, rational individuals (high in need for cognition) tend to engage in conscious aesthetic estimations. However, intuitive individuals (low in need for cognition) are more prone to make emotional estimations (Wu et al., 2022). Top-down attention, affected by factors such as an individual’s motivation and involvement, is usually consciousness-oriented and requires cognitive costs (Banerjee et al., 2015), while bottom-up attention is affected by stimulus characteristics that are usually automated and unconscious (Tu et al., 2013). Liechty et al. (2003) found that the distribution of attention is more affected by bottom-up factors in the environment when consumers search for information without a clear goal, and they tend to show a longer saccade amplitude in the eye movement trajectory (the distance between two consecutive fixations). A shorter saccade amplitude often requires sufficient visual processing resources to analyze and process the information concerned (Rayner, 1998). The distribution of attention is more affected by top-down factors in the environment. The current study further found significant differences between rational thinking individuals and intuitive thinking individuals during information searches, which revealed different cognitive processes for each pair of tasks.

Third, tourists who prefer rational thinking have more positive aesthetic preferences and emotional arousal in the bottom-up process. In contrast, aesthetic preference and emotional arousal showed no significant differences in terms of different thinking styles in the top-down process. In summary, the present study verified that aesthetic preference is more likely to be influenced by both thinking style and visual attention processing. Previous studies suggest that visual saliency influences choices when individuals do not have strong preferences for options (a freely browsing task; Milosavljevic et al., 2012), or when more visually salient options receive more attention and are more likely to be chosen (Luo et al., 2022). The effort to understand complex images results in content that is more detailed and evocative, thus generating greater affection (Sparks and Wang, 2014). In addition, Ares et al. (2014) believed that consumers who rely on rational thinking can conduct more in-depth information processing than consumers who rely on intuitive thinking. Consumers with rational thinking need to search deeper and longer for information and conduct deeper analysis before making their decisions. Therefore, tourists who mainly use rational thinking modes and those who rely on intuitive thinking modes exhibit different information processing methods when processing landscape picture information. Tourists with a highly cognition-related rational thinking style are particularly overt in terms of visual preference and arousal.

### Theoretical implications

First, the CEST has been widely used in marketing and psychology. The differences in people’s dependence on thinking modes and methods of using these modes will affect the evaluation and decision-making of different types of information. However, individual thinking differences in assessing the preferences for and emotions induced by natural and built environments are not well understood. In this study, eye movement characteristics of natural and built environments were verified to be different under two kinds of thinking styles through eye-tracking experiments. This study improves the theoretical justification according to previous studies on the differences in the effects of different thinking styles on environmental aesthetic preferences and emotions. At the same time, tourists’ aesthetic preferences may differ due to different visual attention processes, which has not been clearly confirmed in previous studies.

Second, this study extends the research perspective of visual marketing attention theory, combines previous research results (e.g., visual marketing attention theory and CEST theory), studies whether top-down or bottom-up attention influences individuals with different thinking types, and a new perspective for studying the visual processing characteristics of tourism consumers.

### Practical implications

First, because visitors are increasingly exposed to visual materials, their preference becomes extremely important. Pictures are a major method that attracts tourists. Therefore, travel destination managers should lay more emphasis on the preferences of photos. This study provides insights into image preferences, which may help travel marketers form more effective picture stimuli. In other words, tourists and destination managers can co-create the destination images, which make more easier to attract tourists. Moreover, for attracting tourists, travel destination staff can embellish photos to make tourists feel relaxed and arousing. For built environment sceneries, regulators can add some natural elements to the layout, which may increase tourists’ arousal and stimulate their willingness to visit. For example, hotels can attract tourists by presenting architectural styles with a natural environment layout appended to attract more tourists.

Second, our findings demonstrate the relationship between visual marketing attention theory and CEST. This study suggests that travel destination managers develop targeted publicity strategies for tourists with different thinking styles. For example, detailed tourist brochures (rational individuals and top-down) and visual propaganda materials with higher preferences (intuitive individuals and bottom-up). Individualized services are more easily accepted by tourists. If tourists’ positive inner emotions are triggered, their positive evaluation of the destination will attract new as well as repeat visitors.

### Limitations and future research

This study has several limitations. First is the small sample size of eye tracking, mainly limited by experimental conditions and costs. Second, this study examines how tourists’ thinking styles influence their preferences and arousal in a given situation. Owing to the specific context, tourists’ thinking styles may become intuitive or rational. In other words, situation-specificity could affect tourists’ thinking styles, which this experiment may activate. Even if one tourist shows an intuitive thinking tendency in one study, he may become more rational in a different situation. In future studies, the experimental conditions should not be intentionally controlled. In addition, a control experiment must be conducted to reduce the influence of decision-making scenarios. Finally, future research should consider more types and larger samples. In addition, the potential role of sustainability and digital transition issues as perspective of analysis (e.g., how does the use of digital technology influence the consumption behavior of tourists) in discussing the future research directions.

## Data availability statement

The original contributions presented in the study are included in the article/supplementary material, further inquiries can be directed to the corresponding author.

## Ethics statement

The studies involving human participants were reviewed and approved by the Ethics Committee of School of Humanities and Social Sciences at Fuzhou University. The patients/participants provided their written informed consent to participate in this study.

## Author contributions

WC and WD: conceptualization. WD: methodology, resources, writing—review and editing, project administration, and funding acquisition. RR: software, formal analysis, investigation, data curation, writing—original draft preparation, and visualization. JG: supervision. WC, RR, and WD: validation. All authors contributed to the article and approved the submitted version.

## Funding

The research was supported by the National Social Science Foundation of China (Grant No. 19ZDA043).

## Conflict of interest

The authors declare that the research was conducted in the absence of any commercial or financial relationships that could be construed as a potential conflict of interest.

## Publisher’s note

All claims expressed in this article are solely those of the authors and do not necessarily represent those of their affiliated organizations, or those of the publisher, the editors and the reviewers. Any product that may be evaluated in this article, or claim that may be made by its manufacturer, is not guaranteed or endorsed by the publisher.

## References

[ref1] Alaybek B. Wang Y. Dalal R. S. Dubrow S. Boemerman L. S. G. (2022). The relations of reflective and intuitive thinking styles with task performance: a meta-analysis[J]. Pers. Psychol. 75, 295–319. doi: 10.1111/peps.12443

[ref2] Alghowinem S. AlShehri M. Goecke R. Wagner M. (2014). Exploring eye activity as an indication of emotional states using an eye-tracking sensor. Stud. Comput. Intell. 542, 261–276. doi: 10.1007/978-3-319-04702-7_15

[ref3] Ares G. Mawad F. Giménez A. Maiche A. (2014). Influence of rational and intuitive thinking styles on food choice: preliminary evidence from an eye-tracking study with yogurt labels. Food Qual. Prefer. 31, 28–37. doi: 10.1016/j.foodqual.2013.07.005

[ref4] Banerjee S. Frey H. P. Molholm S. Foxe J. J. (2015). Interests shape how adolescents pay attention: the interaction of motivation and top-down attentional processes in biasing sensory activations to anticipated events[J]. Eur. J. Neurosci. 41, 818–834. doi: 10.1111/ejn.12810, PMID: 25546318 PMC6287492

[ref5] Bigne E. Chatzipanagiotou K. Ruiz C. (2020). Pictorial content, sequence of conflicting online reviews and consumer decision-making: the stimulus-organism-response model revisited. J. Bus. Res. 115, 403–416. doi: 10.1016/j.jbusres.2019.11.031

[ref6] Boardman R. Mccormick H. (2021). Attention and behaviour on fashion retail websites: an eye-tracking study[J]. Inf. Technol. People. doi: 10.1108/ITP-08-2020-0580

[ref7] Brunyé T. T. Gardony A. L. (2017). Eye tracking measures of uncertainty during perceptual decision making[J]. Int. J. Psychophysiol. 120, 60–68. doi: 10.1016/j.ijpsycho.2017.07.008, PMID: 28732659

[ref8] Campos A. C. Pinto P. Scott N. (2020). Bottom-up factors of attention during the tourist experience: an empirical study[J]. Curr. Issue Tour. 23, 3111–3133. doi: 10.1080/13683500.2019.1681383

[ref9] Cui W. Q. Guo X. M. Lu T. T. Hou S. M. (2020). A study on visual attractiveness evaluation method of commercial street based on visual perception: a case study of main street in Huanggaihua town [C]. In National Conference on Architectural Digital Technologies in Education and Research, pp. 246–252.

[ref10] Deng W. Lin Y. Chen L. (2021). Exploring destination choice intention by using the tourism photographic: from the perspectives of visual esthetics processing[J]. Front. Psychol. 5220 10.3389/fpsyg.2021.713739 PMC8636597 34867592

[ref11] Epstein S. Pacini R. Denes-Raj V. Heiser H. (1996). Individual differences in intuitive-experiential and analytical-rational thinking styles. J. Pers. Soc. Psychol. 71, 390–405. doi: 10.1037/0022-3514.71.2.390, PMID: 8765488

[ref12] Folk C. L. Remington R. W. Johnston J. C. (1992). Involuntary covert orienting is contingent on attentional control settings[J]. J. Exp. Psychol. Hum. Percept. Perform. 18, 1030–1044. PMID: 1431742

[ref13] Kahneman D. (2003). Maps of bounder rationality: psychology for behavioral economics. Am. Econ. Rev. 93, 1449–1475. doi: 10.1257/000282803322655392

[ref14] Kinner V. L. Kuchinke L. Dierolf A. M. Merz C. J. Otto T. Wolf O. T. (2017). What our eyes tell us about feelings: tracking pupillary responses during emotion regulation processes. Psychophysiology 54, 508–518. doi: 10.1111/psyp.12816, PMID: 28072452

[ref15] Liechty J. Pieters R. Wedel M. (2003). Global and local covert visual attention: evidence from a Bayesian hidden Markov model. Psychometrika 68, 519–541. doi: 10.1007/BF02295608

[ref16] Lin S. W. Lo L. Y. S. (2016). Evoking online consumer impulse buying through virtual layout schemes. Behav. Inform. Technol. 35, 38–56. doi: 10.1080/0144929X.2015.1056546

[ref17] Luo S. Lin H. Hu Y. Fang C. (2022). Preliminary study on the aesthetic preference for taillight shape design[J]. Int. J. Ind. Ergon. 87:103240. doi: 10.1016/j.ergon.2021.103240

[ref18] Mano H. Oliver R. L. (1993). Assessing the dimensionality and structure of the consumption experience: evaluation, feeling, and satisfaction. J. Consum. Res. 20, 451–466. doi: 10.1086/209361

[ref19] Milosavljevic M. Navalpakkam V. Koch C. Rangel A. (2012). Relative visual saliency differences induce sizable bias in consumer choice[J]. J. Consum. Psychol. 22, 67–74. doi: 10.1016/j.jcps.2011.10.002

[ref20] Motoki K. Saito T. Onuma T. (2021). Eye-tracking research on sensory and consumer science: a review, pitfalls and future directions[J]. Food Res. Int. 145:110389. doi: 10.1016/j.foodres.2021.110389, PMID: 34112392

[ref21] Nagai Y. Georgiev G. V. (2011). The role of impressions on users’ tactile interaction with product materials: an analysis of associative concept networks[J]. Mater. Des. 32, 291–302. doi: 10.1016/j.matdes.2010.05.040

[ref22] Orquin J. L. Mueller L. S. (2013). Attention and choice: a review on eye movements in decision making. Acta Psychol. 144, 190–206. doi: 10.1016/j.actpsy.2013.06.003, PMID: 23845447

[ref23] Phillips W. J. Fletcher J. M. Marks A. D. G. Hine D. W. (2016). Thinking styles and decision making: a meta-analysis[J]. Psychol. Bull. 142, 260–290. doi: 10.1037/bul0000027, PMID: 26436538

[ref24] Pieters R. Wedel M. (2004). Attention capture and transfer in advertising: brand, pictorial, and text-size effects[J]. J. Mark. 68, 36–50. doi: 10.1509/jmkg.68.2.36.27794

[ref25] Rayner K. (1998). Eye movements in reading and information processing: 20 years of research. Psychol. Bull. 124, 372–422. doi: 10.1037/0033-2909.124.3.372, PMID: 9849112

[ref26] Scott N. Zhang R. le D. Moyle B. (2019). A review of eye-tracking research in tourism[J]. Curr. Issue Tour. 22, 1244–1261. doi: 10.1080/13683500.2017.1367367

[ref27] Sparks B. A. Wang Y. (2014). Natural and built photographic images: preference, complexity, and recall[J]. J. Travel Tour. Mark. 31, 868–883. doi: 10.1080/10548408.2014.890155

[ref28] Straffon L. M. Agnew G. Desch-Bailey C. van Berlo E. Goclowska G. Kret M. (2022). Visual attention bias for self-made artworks[J]. Psychol. Aesthet. Creat. Arts. doi: 10.1037/aca0000451

[ref29] Tamás B. Barta A. Szamosközi I. (2021). The role of stimuli complexity and handedness on visual symmetry and asymmetry preference[J]. Eur. J. Behav. Sci. 4, 35–41. doi: 10.33422/ejbs.v4i2.594

[ref30] Tractinsky N. (1997). “Aesthetics and apparent usability: empirically assessing cultural and methodological issues” in Proceedings of ACM SIGCHI (New York, NY: ACM), 115–122.

[ref31] Tu S. Qiu J. Martens U. Zhang Q. (2013). Category-selective attention modulates unconscious processes in the middle occipital gyrus[J]. Conscious. Cogn. 22, 479–485. doi: 10.1016/j.concog.2013.02.007, PMID: 23518233

[ref32] Twedt E. Rainey R. M. Proffitt D. R. (2019). Beyond nature: the roles of visual appeal and individual differences in perceived restorative potential[J]. J. Environ. Psychol. 65:101322. doi: 10.1016/j.jenvp.2019.101322

[ref33] Van der Lans R. Wedel M. (2017). “Eye movements during search and choice” in Handbook of Marketing Decision Models. eds. Wierenga B. van der Lans R. (Cham: Springer), 331–359.

[ref34] Wang Y. Highhouse S. Lake C. J. Petersen N. L. Rada T. B. (2017). Meta-analytic investigations of the relation between intuition and analysis[J]. J. Behav. Decis. Mak. 30, 15–25. doi: 10.1002/bdm.1903

[ref35] Wang Y. Sparks B. A. (2016). An eye-tracking study of tourism photo stimuli: image characteristics and ethnicity. J. Travel Res. 55, 588–602. doi: 10.1177/0047287514564598

[ref36] Wang T. C. Tsai C. L. Tang T. W. (2019). Restorative quality in tourist hotel marketing pictures: natural and built characteristics. Curr. Issue Tour. 22, 1679–1685. doi: 10.1080/13683500.2018.1471051

[ref37] Wedel M. Pieters R. (2008). “A review of eye-tracking research in marketing” in Review of Marketing Research. ed. Malhotra N. K. (Bingley, West Yorkshire: Emerald Group), 123–147.

[ref38] Wedel M. Pieters R. (2017). A review of eye-tracking research in marketing[J]. Rev. Mark. Res. 4, 123–147. doi: 10.4324/9781351550932-5

[ref39] Witteman C. van den Bercken J. Claes L. Godoy A. (2009). Assessing rational and intuitive thinking styles[J]. Eur. J. Psychol. Assess. 25, 39–47. doi: 10.1027/1015-5759.25.1.39

[ref40] Wu Y. Kuru O. Campbell S. W. Baruh L. (2022). Explaining health misinformation belief through news, social, and alternative health media use: the moderating roles of need for cognition and faith in intuition[J]. Health Commun., 1–14. doi: 10.1080/10410236.2021.2010891, PMID: 34978236

[ref41] Xu Y. Shen R. (2022). Aesthetic evaluation of Chinese calligraphy: a cross-cultural comparative study[J]. Curr. Psychol., 1–14. doi: 10.1007/s12144-022-03390-7

[ref42] Zhou L. Zhang Y. Y. Wang Z. J. Rao L. L. Wang W. Li S.. (2016). A scanpath analysis of the risky decision-making process[J]. J. Behav. Decis. Mak. 29, 169–182. doi: 10.1002/bdm.1943

